# One-step fabrication of porous GaN crystal membrane and its application in energy storage

**DOI:** 10.1038/srep44063

**Published:** 2017-03-10

**Authors:** Lei Zhang, Shouzhi Wang, Yongliang Shao, Yongzhong Wu, Changlong Sun, Qin Huo, Baoguo Zhang, Haixiao Hu, Xiaopeng Hao

**Affiliations:** 1 State Key Lab of Crystal Materials, Shandong University, Jinan, 250100, P.R. China

## Abstract

Single-crystal gallium nitride (GaN) membranes have great potential for a variety of applications. However, fabrication of single-crystalline GaN membranes remains a challenge owing to its chemical inertness and mechanical hardness. This study prepares large-area, free-standing, and single-crystalline porous GaN membranes using a one-step high-temperature annealing technique for the first time. A promising separation model is proposed through a comprehensive study that combines thermodynamic theories analysis and experiments. Porous GaN crystal membrane is processed into supercapacitors, which exhibit stable cycling life, high-rate capability, and ultrahigh power density, to complete proof-of-concept demonstration of new energy storage application. Our results contribute to the study of GaN crystal membranes into a new stage related to the elelctrochemical energy storage application.

Gallium nitride (GaN) has become one of the most promising semiconductors because of its excellent properties, which include wide direct bandgap, high thermal stability, excellent electron velocities, and superior chemical and physical stabilities^1^,^2^. With these properties, GaN has potential for a wide range of applications in manufacturing next-generation optoelectronics and high-power and high-frequency devices^3^. Single crystal GaN membranes have recently attracted much attention because of its unique electronic, optoelectronic, and mechanical properties^4^,^5^,^6^,^7^. GaN membrane-based light-emitting diodes^4^, normally off enhancement-type GaN membrane metal oxide semiconductor transistors^5^, and GaN membrane-based flexible optoelectronic devices^6^ have been fabricated. These devices exhibit excellent performance, especially in terms of energy storage; n-type single-crystal GaN porous membrane was used as electrode of the supercapacitor, which exhibits excellent cycling lifespan and ultrahigh power density^7^. GaN crystal membranes offer many superior features that cannot be reproduced in other material forms; they are of central importance to a rapidly expanding frontier^8^.

Significant efforts have been made to prepare GaN membranes, including laser^9^, chemical^10^, electrochemical^4^,^5^,^6^,^11^, and mechanical liftoff on suitable buffer layers^12^,^13^. Despite these developments however, established methods contain multiple steps and sometimes require utilization of expensive equipment, which greatly hinder the further development of GaN crystal membranes in practical application. Our previous work fabricated single-crystal GaN membranes using two-step electrochemical etching^7^. However, the two-step electrochemical etching technique is complex and easily introduce contaminations. Therefore, developing simple non-polluting techniques for preparing GaN crystal membranes is a critical problem.

GaN decomposes when heated at high temperatures (>900 °C)^14^,^15^. The hetero-epitaxy GaN films have high-density dislocations, and decomposition can occur at some dislocation sites to form small V shaped pits^16^,^17^. The formation of V shaped pits can be explained by Cabrera’s thermodynamic theory (Supporting information, Equations S1S2S3S4S5S6,S7)^16^,^18^, based on which we designed and fabricated large-area (≥10 mm × 10 mm) porous GaN crystal membranes (GaNPM) using a one-step high-temperature annealing technique. No other studies have reported on high-temperature annealing technique to fabricate GaN membranes. The GaNPM-based supercapacitors are fabricated and exhibit excellent electrochemical properties, which also proved that GaNPM is a potential supercapacitor electrode material that can be applied to high-power urgent electrochemical energy storage.

## Results and Discussion

### Separation mechanism and process of GaNPM

**Figure 1:**
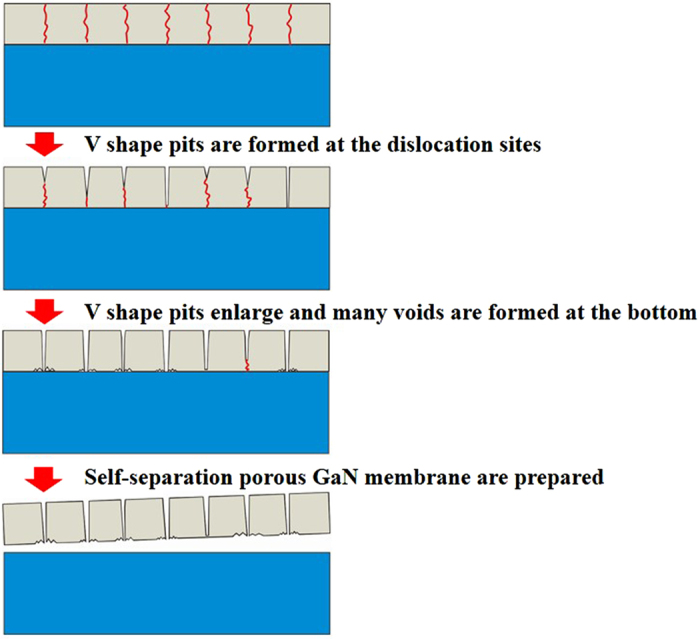
Schematic diagram of separation model to fabricate GaNPM.

Figure 1 shows the schematic diagram of the separation model to fabricate GaNPM. Three steps fabricate the GaNPM. First, V shaped pits are formed at the dislocation sites. Next, the little V shaped pits decompose towards the dislocation and reach the interface of GaN and sapphire as annealing time increases. Finally, the bottom of V shaped pits increases, and many voids are formed on the backside of the GaN layer along with time. Separation occurs at the interface of the sapphire and porous GaN layer. This proposed model is used to explain formation of the GaNPM, which is observed for the first time.

**Figure 2:**
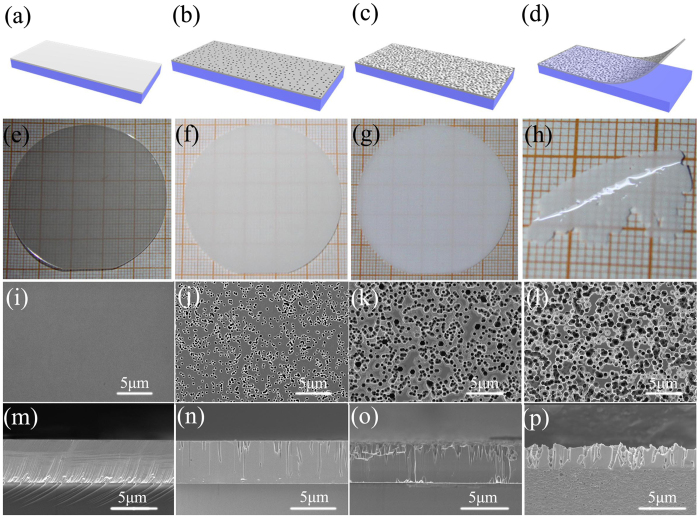
(**a–d**) Schematic illustration of the fabrication process of GaNPM; (**e–h**) surface and (**i–l**) cross-section SEM images of different stages of the GaNPM formation.

Figure 2a to d show the schematic illustration of the fabrication process of GaNPM. Figure 2e to h show the photos of GaN at different stages for GaNPM formation. Figure 2i to l and 2m to p show the surface and cross-section SEM images of GaN at different stages for the GaNPM formation, respectively. The surface of as-grown GaN is smooth and shows no pores on the surface and cross section (Fig. 2a,e,i and m). As annealing time increases (1200 °C, 30 min), many pores appear, and a porous structure is formed (Fig. 2b,f,j and n). The mean diameter of pores is approximately 200 nm. After annealing for 60 min at 1200 °C, the size of pores increased to 500 nm, and many voids have formed at the bottom of the GaN layer (Fig. 2c,g,k and o). Some parts the GaN layer become attached to the sapphire substrate, while some are separated from it (Supporting information, Figure S1). When sintering time increased to 80 min at 1200 °C, large area and self-separation GaNPM is formed (Fig. 2d,h,l and p; Supporting information, Figure S2). The pores are distributed evenly over the GaN membrane. The porosity of GaNPM is approximately 70% (porosity = pore density × single pore area/unit area × 100%). The diameter of the pores is approximately 500–800 nm. The thickness of GaNPM is uniform at about 3 μm.

**Figure 3:**
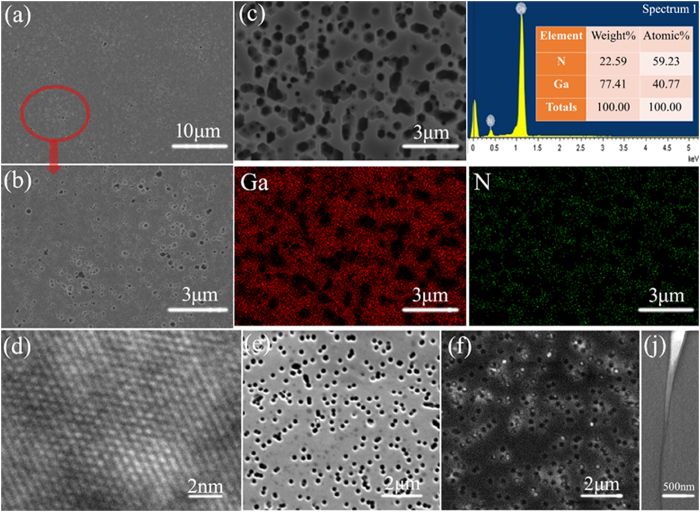
(**a–b**) SEM images of GaNPM backside under high and low magnification, (**c**) energy dispersive X-ray spectroscopy (EDS) element mapping of GaNPM, (**d**) high-resolution transmission electron microscopy (HRTEM) image of GaNPM, (**e**) secondary electron (SE) image, (**f**) cathodoluminescence image of porous GaN samples (1200 °C for 30 min) and (**j**) cross-sectional TEM image of V shaped pit.

Figure 3a,b show the backside SEM images of GaNPM under high and low magnification. Figure S3 shows the tilt-view backside and cross-sectional SEM images of the free-standing GaNPM under high and low magnifications. Many pores can be observed on the backside of GaNPM, which suggests that the pores make an essential contribution to the separation process. Figure 3c shows the results of the EDS mapping of GaNPM. The composition of Ga and N in GaNPM was confirmed by EDS mapping analyses. An HRTEM image (Fig. 3d) confirmed the crystallinity of the self-separation GaNPM. The axis of imaging was aligned to the (001) plane. The lattice fringes obtained from the HRTEM image confirm that the self-separation GaNPM has a high-quality single-crystal structure. The crystal plane spacing of self-separation GaNPM is approximately 0.278 nm, which corresponds to (100) interplanar spacing (0.276 nm) of hexagonal GaN single crystals^19^. A SE image and a cathodoluminescence image of porous GaN (annealed at 1200 °C for 30 min) are presented in Fig. 3e,f. The many dark dots in Fig. 3f are relate to the non-radiative carrier recombination at dislocations. These dark dots in GaN are localized strongly given the short-hole carrier diffusion length^20^. The density of the dark spots is approximately 3 × 10^8^ cm^−2^. The dislocation density of GaNAG is about 3 × 10^8^ cm^−2^ (Supporting information, Figure S4). The density and position of the dark spot are almost the same as the V shaped pits, which also indicate that the decomposition occurred at dislocation sites first to form V shaped pits. Figure 3j shows a threading dislocation at the bottom of the V shaped pit. Moreover, V shaped pits are formed toward the dislocation as predicted from the proposed model.

### Structure and optical properties of GaNPM

**Figure 4:**
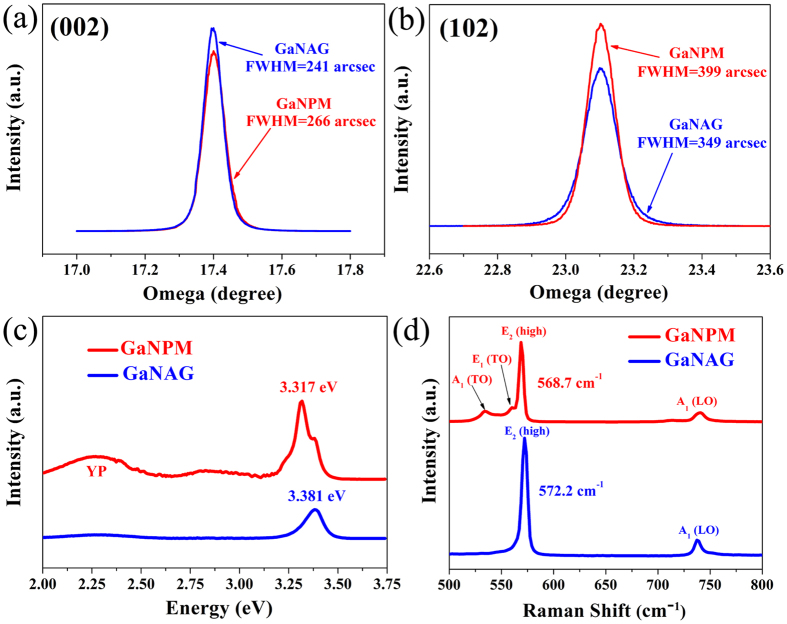
GaNPM and GaNAG structure characterization by (**a,b**) HRXRD rocking curves of GaNPM and GaNAG (002) ω-scans and (102) ω-scans; (**c**) photoluminescence (PL) spectra; (**d**) Raman spectroscopy of GaNPM and GaNAG samples.

Figure 4a,b show the ω scan spectra of (002) and (102) planes of GaNAG and GaNPM, respectively. For the (002) peak of GaNAG and GaNPM, the full width at half-maximum (FWHM) values are 241 and 266 arcsec, respectively. For the (102) peak of GaNAG and GaNPM, the FWHM values are 349 and 399 arcsec, respectively. The GaNPM sample exhibits larger peak width than that of the (002) and (102) diffraction planes of the GaNAG sample. The reason for this result has been explained in previous reports^5^,^21^: The GaNPM become thinner compared with GaNAG. The results also confirm the high crystal quality of GaNPM.

PL measurements were carried out on GaNAG and self-separation GaNPM to characterize the optical properties. Figure 4c shows that the band edge emissions of GaNAG and GaNPM could be observed at 3.381 and 3.317 eV, respectively. The band edge emission peaks of GaNPM were slightly red-shifted relative to the GaNAG because of the relaxation of the compressive stress typically present for GaNAG. The intensity of the band-edge emission for self-separation GaNPM was enhanced by a factor of 2 because of the light scattering of pores in GaN^22^. Yellow emission peaks (YP), which are related to the defects around 2.0–2.6 eV, were observed for self-separation GaNPM. The YP can be attributed to native defects, such as vacancies, N defects, interstitials and anti-sites^23^,^24^. The porous structure may result in the formation of these defects, which caused a noticeable improvement in the YP. The additional side peak on the flank (3.381 eV) in GaNPM suggests that some parts of the GaN membrane are not porous, and do not relax. Thus the band gap of GaN is maintained.

Raman scattering assesses the microscopic disorder and strain state of GaN by measuring the frequency, polarization properties and broadening of the Raman active phonons. Figure 4d shows the Raman spectroscopy of self-separation GaNPM and GaNAG. Raman spectroscopy is obtained under z (xx) −z back-scattering geometry. According to Raman selection rules, the two allowed phonon modes in this scattering geometry are E_2_ (high) and A_1_ (LO)^25^. The A_1_ (LO) and E_2_ (high) peaks can be observed in the GaNAG and self-separation GaNPM. In addition to the GaN A_1_ (LO) and E_2_ (high) peaks, forbidden A_1_ (TO) and E_1_ (TO) peaks in a backscattering configuration were also observed in the self-separation GaNPM. The existence of forbidden TO modes can be attributed to the scattering of the pores in GaN^26^.

The state of stress for GaN is also investigated using the E_2_ Raman mode, which is highly sensitive to stress^27^. Figure 4d shows a shift of E_2_ (high) peak from GaNAG (572.2 cm^−1^) to GaNPM (568.7 cm^−1^). Approximately 568 cm^−1^ is the E_2_ (TO) mode position of stress-free GaN^25^, which confirms an almost strain-free state of self-separation GaNPM (568.7 cm^−1^). The relaxation of residual stress can be calculated by equations as follows^28^:


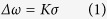


where Δω is the shift of the E_2_ (high) phonon peak, *K* (=4.3 cm^−1^ GPa^−1^) is the proportionality factor, and σ is the in-plane biaxial stress. According to this equation, a compressive stress of approximately 0.977 GPa which exists in GaNAG, can be obtained. The stress relaxation is about 0.814 GPa for GaNPM. The result is consistent with that of the PL result.

The electrical properties of GaNAG and GaNPM were tested using the Hall effect measurement, as shown in Table S1 (Supporting information). Both samples exhibited n-type conduction. Mobility was 341 cm^2^ V^−1^ s^−1^ in GaNAG and 495 cm^2^ V^−1^ s^−1^ in GaNPM. The dislocation lines became negatively charged, and a space charge formed around it, which scattered the electrons traveling across the dislocations, and reduced mobility^29^. The dislocations were removed because of the decomposition of V shaped pits, which increased the mobility of GaNPM. Carrier concentration was reduced from 9.945 × 10^16^ to 3.409 × 10^16^ cm^−3^ because of the porous structure. The electrical conductivity and electrical resistivity of GaNPM are 2.7S cm^−1^ and 0.3699 Ω cm, respectively. This results indicate that GaNPM has excellent electrical performance.

### Electrochemical characterization

The single crystal GaNPM exhibited good conductivity and porous structure. These advantages render GaNPM a good potential electrode material for energy storage devices. The electrochemical properties were investigated using cyclic voltammetry (CV), galvanostatic charge/discharge (GCD) tests, and electrochemical impedance spectroscopy (EIS) measurement. We replaced gravimetric with areal metric to characterize highly accurate metrics in electrochemical SCs^30^,^31^.

**Figure 5:**
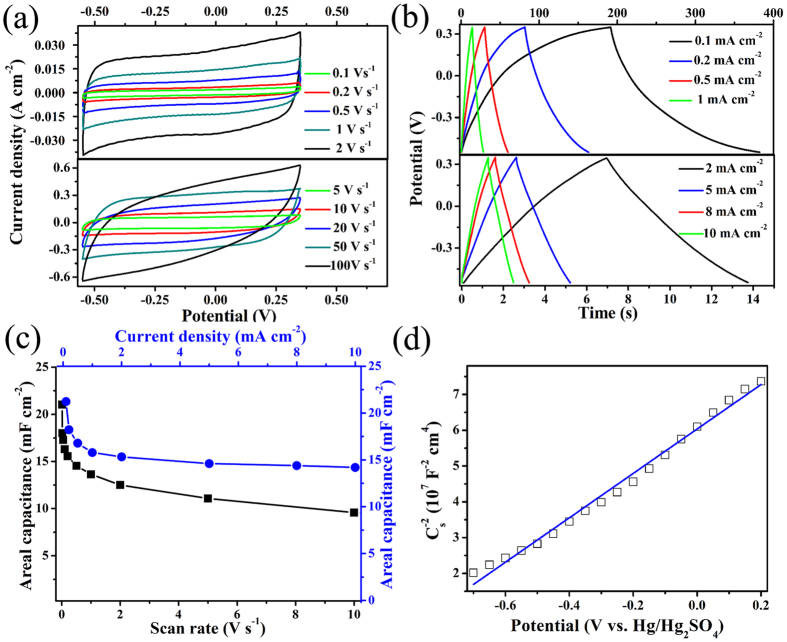
Electrochemical characterization of GaNPM in three-electrode cells: (**a**) CV curves; (**b**) GCD curves; (**c**) specific capacitance at different current density and scan rates; and (**d**) Mott–Schottky plot.

Figures 5a and S5 (Supporting information) show that the CV curves have a quasi-rectangular and symmetric shape with the scan rate ranging from 0.01 V s^−1^ to 100 V s^−1^ of the electrode. This result suggests the presence of a good charge propagation at the electrode/electrolyte interface, following the mechanism of electric double-layer capacitors^32^. GaNPM electrodes manifest the outstanding performance of areal capacitors of 21.05 mF cm^−2^ (scan rate 0.01 V s^−1^) (Supporting information, Figure S1), even maintaining at 13.65 mF cm^−2^ (current density 1 V s^−1^), as shown in Fig. 5c. Many defects are produced during decomposition reactions. Thus, a great many active sites are beneficial to the adsorption of electrolyte ions contribute to the improvement of the electrochemical capacitance of the SCs^27^,^33^.

Figure 5b presents the GCD curves for GaNPM electrodes with current density ranging from 0.1 to 10 mA cm^−2^. All charge/discharge curves were nearly symmetrical with a slight curvature that indicates pseudocapacitive contribution along with the double layer contribution^32^. The areal capacitance of the electrode was 21.22 mF cm^−2^ (current density 0.1 mA cm^−2^) (Fig. 5c), which is higher than that of GaNAG (3.92 mF cm^−2^ at 0.5 mA cm^−2^)^7^. Figure 5c shows that the areal capacitance held at 15.78 mF cm^−2^, which is advanced than some electrode materials, such as CoNiS_2_ (9 mF cm^−2^)^27^, nitride TiO_2_ (1.4 mF cm^−2^ at 1 mA cm^−2^)^34^, SiN (0.11 mF cm^−2^)^35^ and carbon nanotube (CNT)@MnO_2_ (2.43 mF cm^−2^ at 0.5 mA cm^−2^)^36^. When the current density reach up to 10 mA cm^−2^, the capacitance held at 14.22 mF cm^−2^, and the capacity retention of the electrode was approximately 67%, thereby indicating that the single crystal GaNPM has excellent chemical and mechanical stability^37^.

Another important characteristic of high-performance electrode materials is cycling stability. Figure S6 (Supporting information) shows that GaNPM electrode has excellent cycling stability, and capacitance retention rate is about 99% after 10,000 cycles at 5 mA cm^−2^. The initial cycle and after 10,000 cycles of Nyquist plots (Supporting information, Figure S7) almost coincide. The initial and after cycles of series resistance (Rs) is approximately 1 Ω cm^−2^, which indicates that the ion diffusion paths of the electrodes have not been interrupted. This resluts shows that the high mechanical stability of the electrode materials without sacrificing electrochemical performance.

Mott−Schottky (M–S) plots were generated based on capacitances derived from electrochemical impedance obtained at a potential of −0.7 to 0.2 V with 962 Hz frequency (Fig. 5d). The line sections of the plot exhibited a positive slope, thereby demonstrating the n-type feature of semiconductor^38^,^39^,^40^, which is in accordance with Hall measurements in Table S1 (Supporting information).

**Figure 6:**
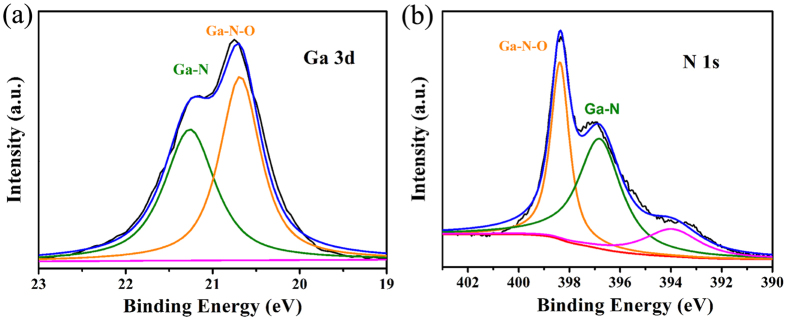
Surface analysis of the GaNPM materials: (**a**) X-ray photoelectron spectroscopy (XPS) spectra of Ga 3d and (**b**) XPS spectra of N 1s.

The X-ray photoelectron spectroscopy (XPS) of the GaNPM materials was measured to explore the pseudocapacitive mechanism further (Fig. 6). The XPS survey spectrum (Supporting information, Figure S8) on the surface of the materials showed that the electrodes were composed of three elements: Ga, N, and O, in accordance with the EDS result mentioned above. The Ga 3d and N 1s high-resolution XPS spectra (Fig. 6a,b) revealed a Ga-N-O bond at 20.7 and 396.8 eV, respectively, which is similar to that of TiN and Nb_3_N_4_^41^,^42^. The N1s spectrum showed a peak at a lower bonding energy (394 eV), which can be attributed to the N defects of the materials^42^. During the decomposition reactions, many vacancies or defects were generated on the surface of pores, which have been proven by PL. These defects and vacancies increased the absorption of electrolyte ion on the surface of the electrode, and further enhanced the capacitance performance under the high rates conditions^7^.

It is reported that the fast energy storage process was improved by the functional groups (oxides) on the surface of the metal nitride with a redox reaction^43^, which is a supplement of the electrical double-layer capacitors (EDLCs). Partly metallic also occur at metal oxynitride thin films with low oxygen contents. Thus, the oxynitride layer may have high electrical conductivity and improve the pseudocapacitance^44^,^45^. Faradic reactions could occur on the surface of oxynitride layer in the H_2_SO_4_ electrolyte, as follows^43^,^46^:


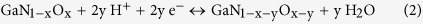


where GaN_1−x−y_O_x−y_ represents the metal oxynitride active layer, the reaction on the right represents the charging process, and the reaction on the left refers to the discharging process.

**Figure 7:**
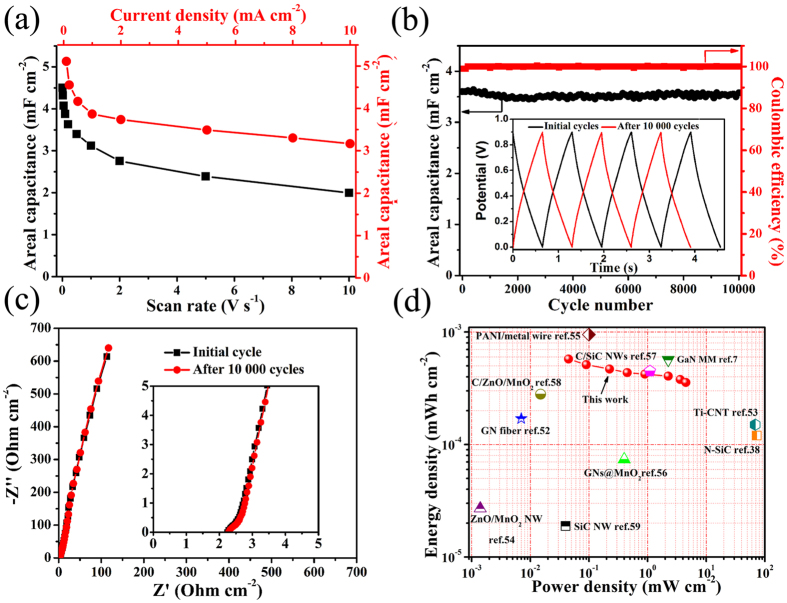
Electrochemical performance tested of GaNPM in two-electrode mode: (**a**) areal capacitance at different current density and scan rates; (**b**) cycling performance at 5 mA cm^−2^, where the inset is GCD curve at the initial stage and after 10,000 cycles; (**c**) Nyquist plots at the initial stage and after 10,000 cycles; and (**d**) areal energy versus power densities compared with the selected previous reports^7^,^37^,^51^,^52^,^53^,^54^,^55^,^56^,^57^,^58^.

We fabricated symmetric supercapacitors and tested its electrochemical performance to explore further the advantages of GaNPM for real applications. The CV curves in Figure S9a (Supporting information) showed an enhanced electrochemical performance with an approximately rectangular CV shape between 0–0.9 V for scan rates from 0.1–100 V s^−1^, which are indicative of nearly ideal capacitive behavior^47^. The cell was also sufficiently robust to be charged/discharged over a broad scan rates (100 V s^−1^) and maintained rectangular CV curve shape. The areal capacitance of the device was 4.5 mF cm^−2^ at a scan rate of 0.1 V s^−1^, and the areal capacitance remained at 81% of this value (3.6 mF cm^−2^, 1 V s^−1^) (Fig. 7a). The superior rate capability of this cell can be attributed to GaN crystal excellent physical and chemical stability.

The GCD curves are collected at a current density in the range of 0.1 to 10 mA cm^−2^ (Supporting information Figure S9b). These data reveal that the charging curves are nearly symmetrical to their discharging counterpart and have good linear voltage-time profiles, which demonstrated good capacitive performance^48^. Figure 7a shows that the areal capacitance of the cell range from 5.11 mF cm^−2^ (0.1 mA cm^−2^) to 3.17 mF cm^−2^ (10 mA cm^−2^), indicating the increasing of a 100-fold current density. The capacitor even holds 62.0%. This outstanding stability of the GaNPM indicated the high electrical conductivity and stable porous structure, which allowed plentiful adsorption of ions and further enhance charge transport and efficient ion adsorption−desorption^49^.

The cycling stability is another important factor that affects the performance of the supercapacitors. This work evaluated the cycling stability of the device for 10,000 cycles (Fig. 7b). No obvious change in cycling performance was observed, thereby confirming the excellent flexibility of the electrochemical device. After 10,000 cycles, areal capacitance was maintained at 3.57 mF cm^−2^, which is close to the initial capacitance of 3.60 mF cm^−2^. Approximately 99% capacitance retention rate was achieved. Typical Nyquist plots of the symmetric supercapacitor for the initial cycle and after 10,000 cycles are shown in Fig. 7c. At a low level, the imaginary part of the impedance sharply increased, and the plots were nearly vertical lines, which are characteristic of capacitive behavior^50^. A smaller semi-circle was obtained at high frequencies, indicating that the R_s_ is extremely low, and is approximately 0.8 Ω cm^−2^. These results also demonstrate that the GaNPM is a kind of very potential electrochemical capacitors electrode material.

Power and energy densities are two other important parameters for evaluating electrochemical performance. The Ragone plot is obtained based on the symmetric supercapacitor, and Fig. 7d shows the calculated values. The maximum areal energy density is 0.58 μW h cm^−2^, and the cell outputted a maximum power density of 45 mW cm^−2^ (10 mA cm^−2^), which indicate the excellent performance of the electrodes. The areal energy-power density of the symmetric supercapacitors are comparable to that of electrical double-layer capacitors (EDLCs), such as graphene (GN) (0.17 μW h cm^−2^, 0.007 mW cm^−2^)^51^, Ti-doped carbon nanotube (CNT) (0.15 μW h cm^−2^, 68.4 mW cm^−2^)^52^; GaN membranes (GaNMM) (0.65 μW h cm^−2^, 45 mW cm^−2^)^7^ as well as pseudocapacitance, such as ZnO/MnO_2_ (0.027 μW h cm^−2^, 0.0014 mW cm^−2^)^53^, polyaniline (PANI)/metal wires (0.95 μW h cm^−2^, 0.1 mW cm^−2^)^54^, N-doped SiC (0.12 μW h cm^−2^, 72.3 mW cm^−2^)^38^, and GN@MnO_2_ (0.074 μW h cm^−2^, 0.4 mW cm^−2^)^55^.

## Conclusions

We have successfully demonstrated the separation of large-area, free-standing, and single-crystalline porous GaN membranes through a one-step high-temperature annealing technique, for the first time. We proposed a model based on the thermodynamic theories to explain the separation process of GaNPM. SEM, TEM, and CL images support the proposed model and demonstrate the complete separation procedure of GaNPM. The GaNPM-based supercapacitors were fabricated and manifested excellent electrochemical characterization (99% capacitance retention after 10000 cycles with symmetric supercapacitors). The cell based on these GaNPM materials also manifested ultrahigh power density (45 mW cm^−2^). These excellent electrochemical performances validate the concept of GaNPM-based supercapacitors and highlight its potential for energy storage application.

## Methods

### Preparation of GaNPM

The MOCVD-GaN/Al_2_O_3_ was used as starting template. Annealing condition was 1200 °C for 30–80 min. After the cooling process, self-separation GaNPM was fabricated.

### Preparation of supercapacitors

In the three-electrode system, the Hg/Hg_2_SO_4_ electrode was used as a reference electrode, a platinum sheet as counter electrode, and 1 M H_2_SO_4_ aqueous solution as electrolyte. The working electrode were fabricated by a stainless steel cloth coated with well-blended slurry. The slurry contained 80 wt.% active material, 10 wt.% poly vinylidene fluoride, and 10 wt.% Super-P in N-methyl-2-pyrrolidone. In a vacuum drying oven, the prepared electrodes were heated for 12 h at 80 °C.

In a two-electrode system, a sulfonation film was used to separate two symmetrical electrodes and a layered structure was assembled. 1 M H_2_SO_4_ aqueous solution was used as electrolyte. The size of active material was about 1 cm^2^ on each electrode.

## Additional Information

**How to cite this article:** Zhang, L. *et al*. One-step fabrication of porous GaN crystal membrane and its application in energy storage. *Sci. Rep.* **7**, 44063; doi: 10.1038/srep44063 (2017).

**Publisher's note:** Springer Nature remains neutral with regard to jurisdictional claims in published maps and institutional affiliations.

## Supplementary Material

Supplementary Information
