# Supplementary material for: One-step fabrication of porous GaN crystal membrane and its application in energy storage
Source: Sci Rep. 2017 Mar 10;7:44063. doi: 10.1038/srep44063 (PMC5345049; doi:10.1038/srep44063)
Supplement: Supplementary Information [file srep44063-s1.pdf]

Supporting Information

One-step fabrication of porous GaN crystal membrane and its application in energy storage

Lei Zhang†, Shouzhi Wang†, Yongliang Shao, Yongzhong Wu, Changlong Sun, Qin Huo, Baoguo Zhang, Haixiao Hu, Xiaopeng Hao*,

State Key Lab of Crystal Materials, Shandong University, Jinan, 250100, P.R. China

*Address correspondence to xphao@sdu.edu.cn

†These authors (Lei Zhang and Shouzhi Wang) contributed equally to this work.

This file includes:

S1. Equations S1-S7

S2.Characterization Methods

S3. Electrochemical Tests

S4. Supplementary Figures S1–S22

S5. Table S1

S1. Equations S1-S7

The GaN decomposition reaction has been reported by Boris V. L'vov to be as follows:[1]

$$GaN\,(s) \rightarrow Ga(g) + (1\text{-}i)N + 0.5iN_2 \qquad (1)$$

where the interaction parameter i varies from 0 to l, depending on the extent to which the nearest nitrogen atoms interact with one another at the instant of decomposition. The morphological reorganization or the corrosion of the GaN material will happen due to the decomposition. The hetero-epitaxy GaN films have high density dislocations, so the decomposition can occur at some

dislocation sites to form small V shaped pits. The formation of V shaped pits can be explained by the Cabrera's thermodynamic theory.[2,3]

The change in the free energy of V shaped pits on a perfect surface is given by

$$\Delta G_p=\Delta G_s+\Delta G_v \qquad (2)$$

where ΔG_s is the change in the surface energy and ΔG_v is the change in the volume energy.

In order to form a V shaped pit nucleus at a perfect surface, an energy should be given as

$$\Delta G_p{}^*=\pi h r_c \gamma= \pi h \gamma^2\ \Omega/\Delta\mu \qquad (3)$$

where h is the depth of V shaped pits nucleus, γ is the edge free energy, r_c is the size of the critical nucleus, Ω is the volume occupied by each atom and Δμ is the potential difference.

The free energy for a V shaped pit nucleation at a dislocation site, ΔG_d, consists of the surface-energy term ΔG_s, the volume-energy term ΔG_v, and a dislocation-energy term E_{disl}. It may be expressed as

$$\Delta G_d=\Delta G_s+\Delta G_v+hE_{disl} \qquad (4)$$

The dislocation energy outside its core is given as

$$E_{dis}=-\ (Gb^2/4\pi)\ \alpha ln(r/r_0) \qquad (5)$$

Where G is the shear modulus, b is the Burgers vector of the dislocation, r0 is the radius of the dislocation core outside which the elastic continuum theory is valid, α=1/(1-v) for a clean dege dislocation and α=1 for a clean screw dislocation (where v is Poisson's ration).

Thus we have

$$\Delta G_d=\Delta Gs+\Delta Gv-\ (Gb^2/4\pi)\ \alpha ln(r/r_0) \qquad (6)$$

The critical free energy for nucleation of a monomolecular V shaped pit at a dislocation is written as

$$\Delta G_d{}^* \cong \Delta G_p{}^*\ (1-4r_F/r_c)^{½} \qquad (7)$$

Where $r_F = \alpha Gb^2/8\pi^2\gamma$ is Frank's radius. According to these equations, it can be obtained that the ΔG_d^* is always smaller than ΔG_p^*. Therefore, the nucleation of V shaped pits occurs preferentially at dislocations.

S2. Characterization Methods

Scanning electron microscopy (SEM) images were taken with a Hitachi S-4800 field emission microscope equipped with a Horiba EX-450 energy-dispersive X-ray spectroscopy (EDS) attachment. The crystalline quality of single-crystal GaN mesoporous membranes (GaNMM) was characterized by high-resolution X-ray diffraction (HRXRD) that utilizes symmetrical (002) and asymmetrical (102) reflections. Raman spectra of samples were obtained with a LabRAM HR system of Horiba Jobin Yvon at room temperature and using a 532 nm solid state laser as the excitation source. High-resolution transmission electron microscopy (HRTEM) images were obtained on a Philips Tecnai Twin-20U high-resolution transmission electron microscope that operates at an accelerating voltage of 200 kV. The transmission electronic microscopy (TEM) specimens of the porous GaN crystal membrane was prepared by conventional method of mechanical polishing followed by ion milling. The sample was imaged in FEI Tecnai TF-20 FEG/TEM operated at 200kV. The cathodoluminescence (CL) experiments were performed in a scanning electron microscope (Hitachi SU-70) equipped with a Gatan Instruments MonoCL3 CL imaging and spectral analysis system. Photoluminescence (PL) measurement was carried out at room temperature using 325 nm He–Cd lasers as the excitation source. Electrical properties were characterized by Hall effect measurement (HL5500 Hall System) at room temperature.

S3. Electrochemical Tests

CV and GCD curves were collected at −0.55 V to 0.35 V against Hg/Hg_2SO_4 for the three-electrode system and 0 V–0.9 V for the two-electrode system by varying the scan rate from

0.1 V s^{-1} to 100 V s^{-1} and current density from 1 mA cm^{-2} to 10 mA cm^{-2}, respectively. Alternating current EIS spectra were collected within a frequency range of 10^{-2} Hz–10^{5} Hz at the open circuit voltage with an AC amplitude of 0.005 V, and Mott–Schottky (M–S) plots were measured at a frequency of 962 Hz.

For three-electrode cells, areal capacitance (C_a) derived from areal discharge curves was calculated from Equation 7, which is represented as[4–6]

$$C_a = \frac{I\Delta t}{S\Delta V} \qquad (7)$$

where C_a is the areal capacitance (F cm^{-2}), I is the constant discharge current (A), Δt is the discharge time (s), ΔV is the voltage window (V), and S is the area of active material on the working electrode (cm^2).

The capacitance for the measured supercapacitor cell (C_{cell}) was calculated from Equation 8, represented as[6, 7]

$$\mathrm{C_{cell}} = \frac{I\Delta t}{S\Delta V} \qquad (8)$$

where s is the total area of the active material on the two electrodes (cm^2).

C_a derived from CVs was calculated from Equation 9, represented as[3, 4]

$$\mathrm{C_a} = \frac{1}{sv(V_b - V_a)}\int_{V_a}^{V_b} I\mathrm{d}V \qquad (9)$$

where s is the area of active material on the working electrode (cm^2), v is the scan rate (V s^{-1}), I is the discharge current (A), V_b and V_a are the high and low voltage limits of the CV curves (V).

The energy density (E) and power density (P) of the supercapacitor cell were estimated according to Equation 10 and 11, respectively, represented as[3–5]

$$\mathrm{E} = \frac{\mathrm{C_{cell}}\Delta V^2}{2} \qquad (10)$$

$$P = \frac{E}{\Delta t} \qquad (11)$$

where ΔV is the cell-operation voltage window (V) and Δt is the discharge time (h).

The electron concentration was calculated according Mott–Schottky (M–S) theory,[8] as in Equation[5]

$$\frac{1}{C_s^2} = \frac{2}{(\varepsilon\varepsilon_0\, e_0 N_d)}\left(U - U_{fb} - \frac{K_B T}{e_0}\right) \qquad (12)$$

where C_s (F cm^{-2}) is the space charge capacitance per unit area, N_d (cm^{-3}) is the electron concentration, ε is the dielectric constant (9 for rutile GaN), ε_0 (F m^{-1}) is the permittivity of vacuum, e_0 (C) is the electron charge, U (V) is the applied potential, U_{fb} (V) is the flat band potential, T (K) is the temperature, and k_B (J K^{-1}) is the Boltzmann constant.

S4. Supplementary Figures S1–S9

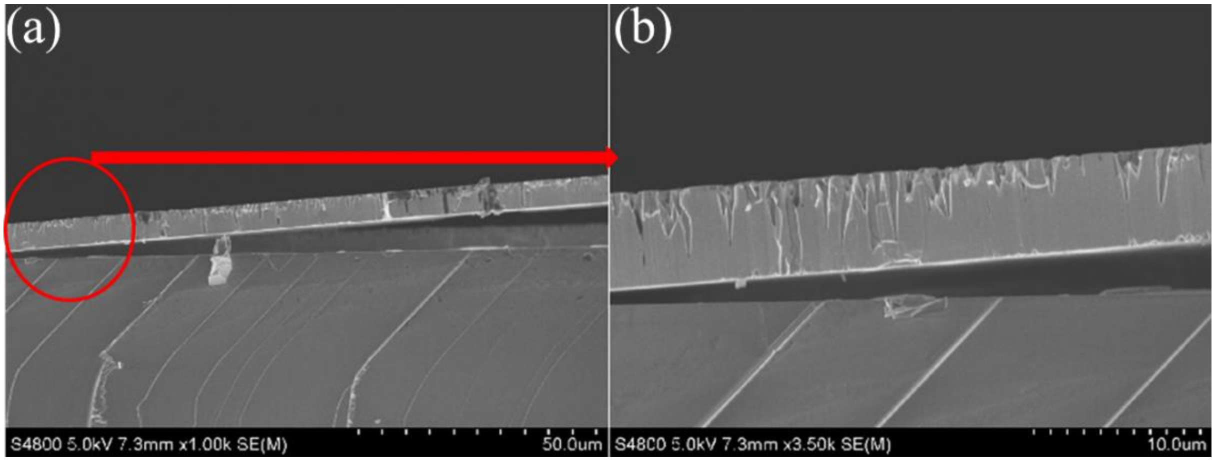

Figure S1. Cross-sectional images of the separating porous GaN crystal membrane (GaNPM) and sapphire substrate under low (a) and high (b) magnification;

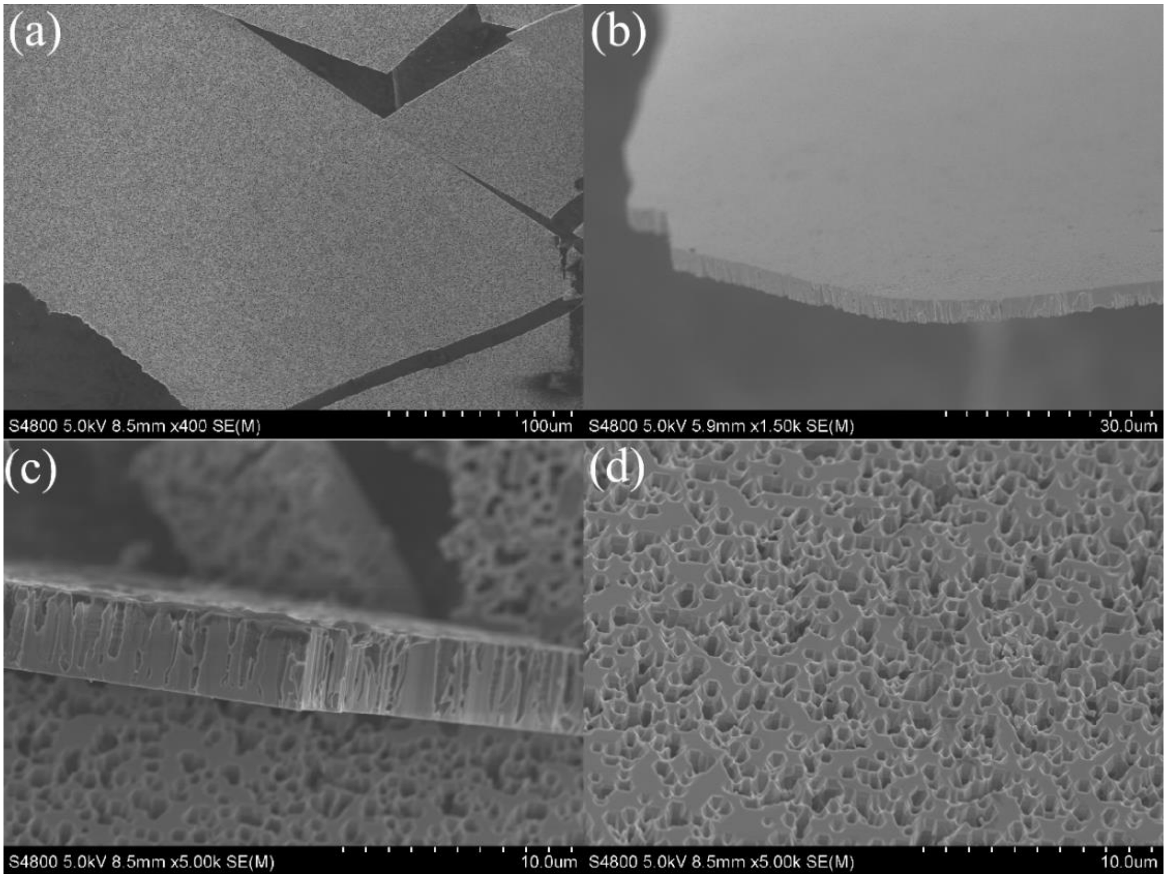

Figure S2. Top-view SEM images of the free-standing GaNPM (a); Tilt-view backside SEM images of the free-standing GaNPM (b); Tilt-view cross-sectional SEM images of the free-standing GaNPM (c); Tilt-view surface SEM images of free-standing GaPM(d)

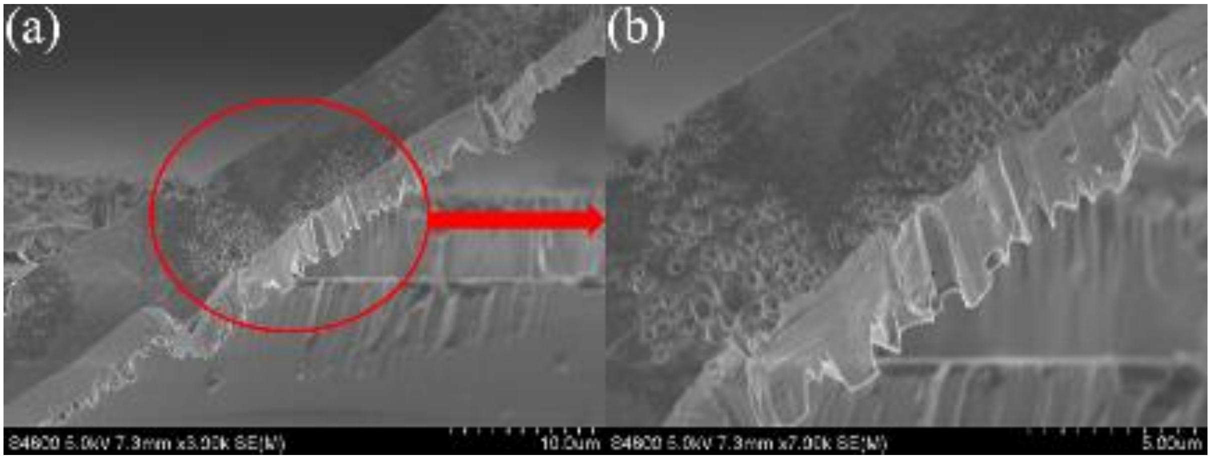

Figure S3. Tilt-view backside and cross-sectional SEM images of the free-standing GaNPM under low (a) and high (b) magnification

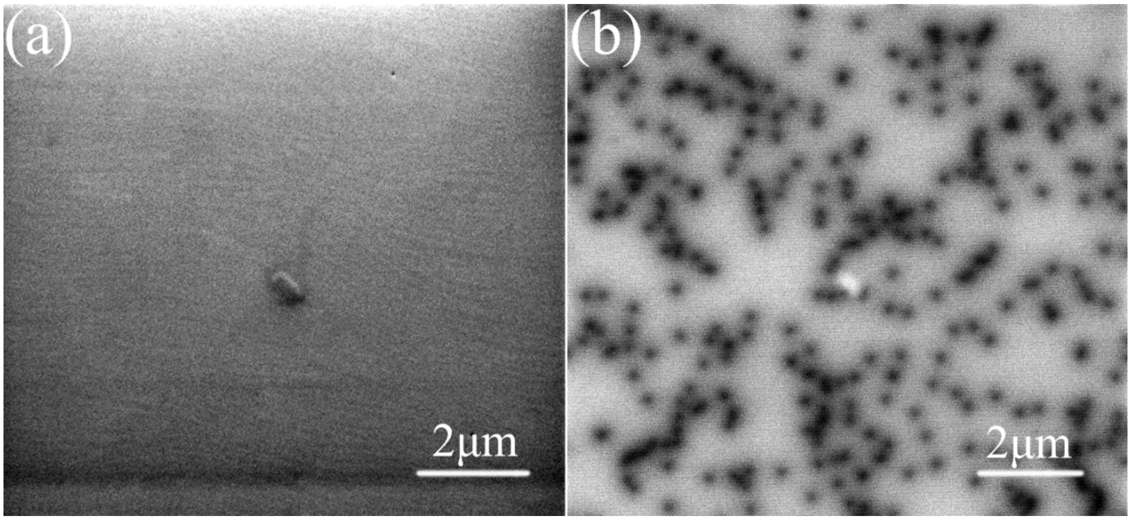

Figure S4. A secondary electron (SE) image (a) and a cathodoluminescence image (b) for the as grown GaN sample.

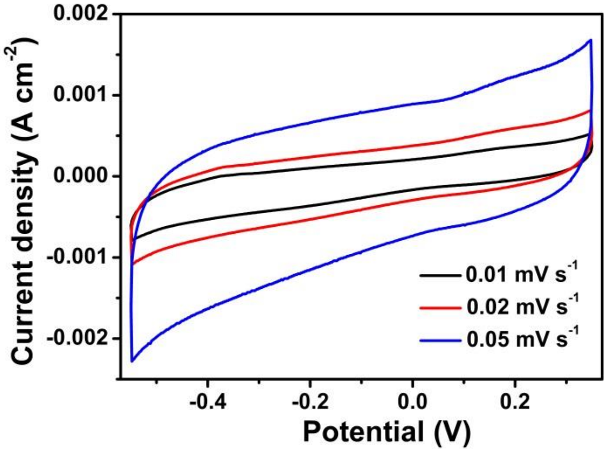

Figure S5.CV profiles of the electrode at scan rates ranging from 0.001 to 0.005 V s^{-1}.

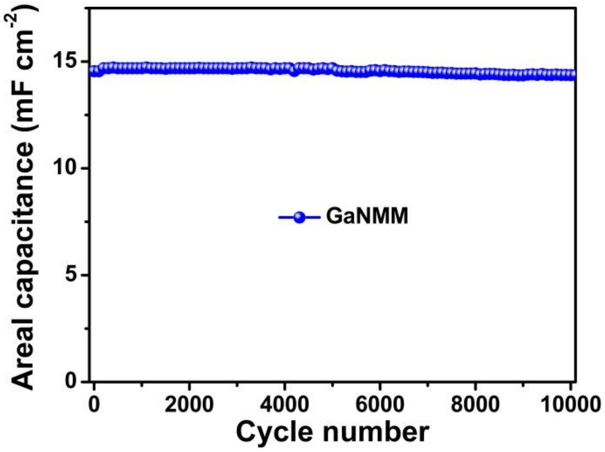

Figure S6. The cycling performance of GaNMM with a current density of 5 mA cm^{-2}.

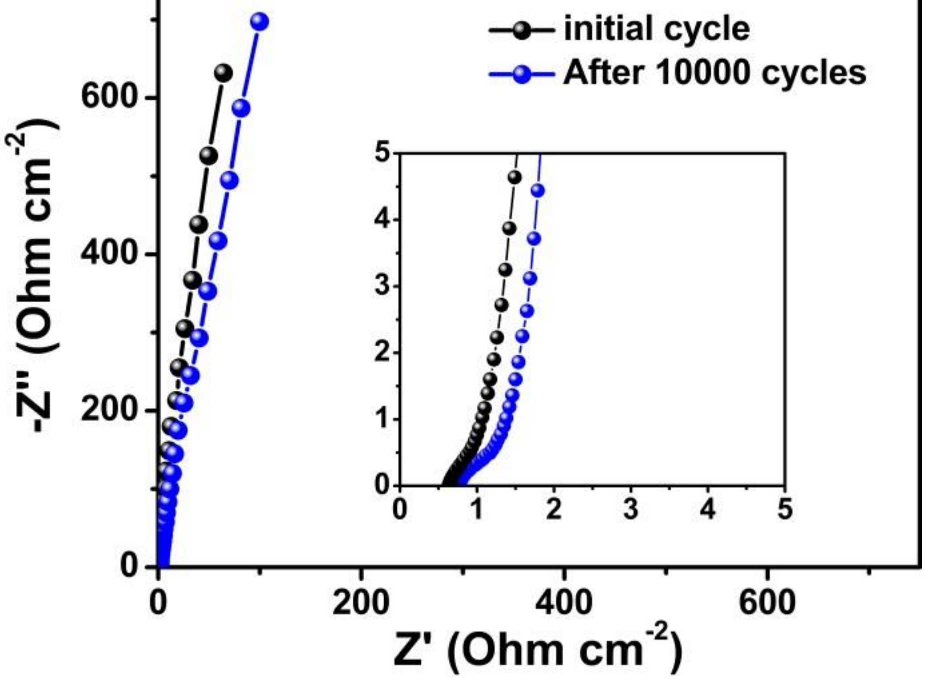

Figure S7. Nyquist plots at the initial stage and after 10,000 cycles of the GaNPM materials.

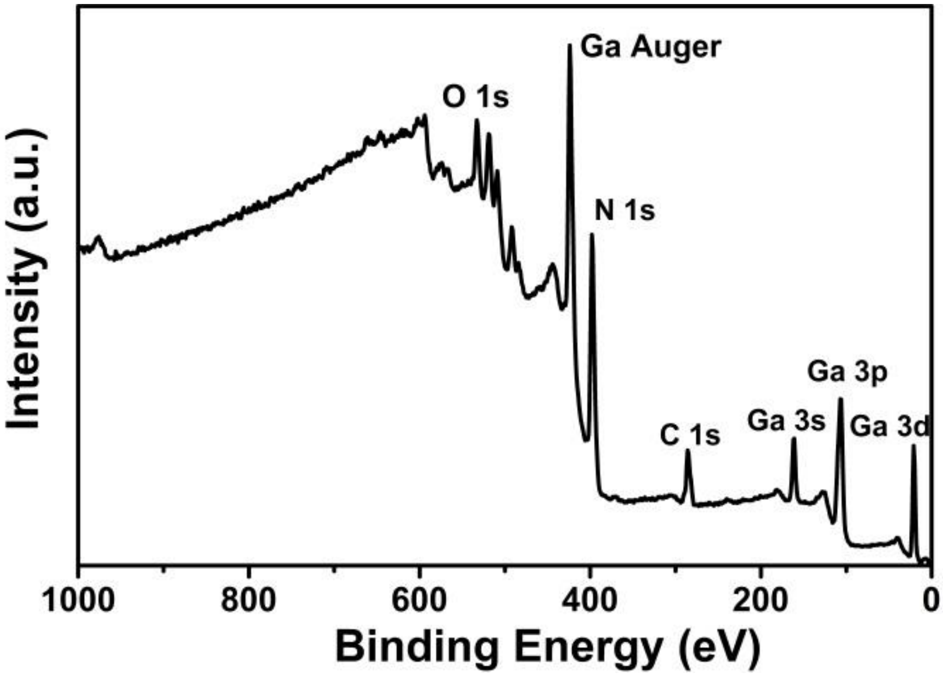

Figure S8. The survey XPS spectrum of GaNPM electrode material.

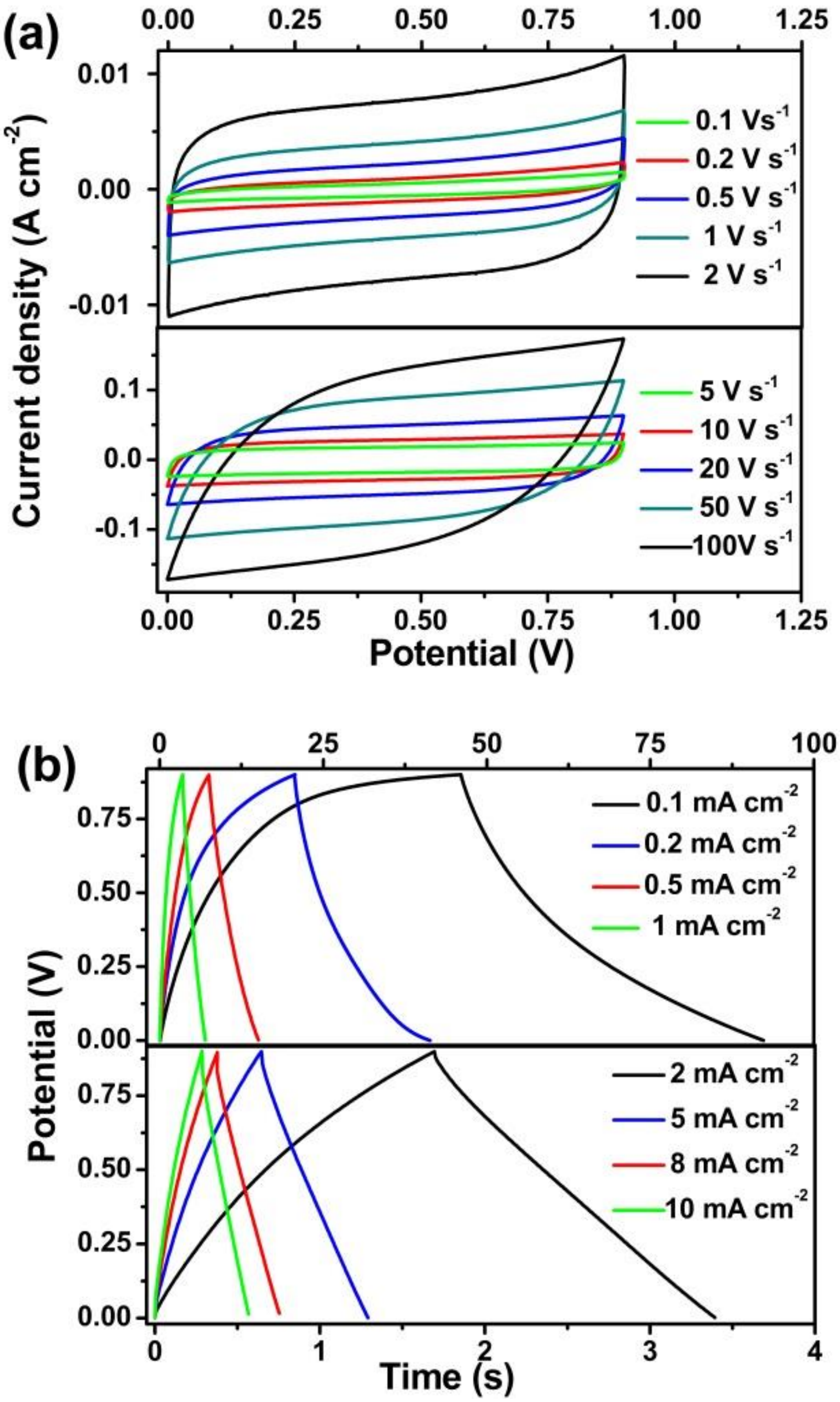

Figure S9. Electrochemical performance tested of GaNPM in two-electrode mode: (a) CV curves at increasing scan rates; (b) GCD profiles at increasing current density.

S5. Table S1

Table S1. Hall effect measurements of sheet resistance (R_{sh}), mobility (μ), carrier concentration (n), electrical resistivity (R), and electrical conductivity (ρ) of GaNAG and GaNPM.

Sample	Type	R_{sh} (Ω sq^{-1})	Mobility (μ) (cm^2 V^{-1} s^{-1})	Carrier concentration (n) (cm^{-3})	R (Ω cm)	ρ (S cm^{-1})
GaNAG	n	368.4	341	9.945×10^{16}	0.1842	5.43
GaNPM	n	739.7	495	3.409×10^{16}	0.3699	2.7

References

1 L'vov, B. V. Kinetics and mechanism of thermal decomposition of GaN. *Thermochim. Acta*, **360**, 85–91 (2000).

2 Sangwal, K. *Etching of crystals—theory, experiment, and application* (eds Amelinckx, S. et al.) Ch. 1, 9-12 (North Holland Physics Publishing, 1987).

3 Tian, Y. et al. Characterization of dislocations in MOCVD-Grown GaN using a high temperature annealing method. *CrystEngComm*, **16**, 2317–2322 (2014).

4 Simon, P. & Gogotsi, Y. Materials for electrochemical capacitors. *Nat. Mater.* **7**, 845-854 (2008).

5 Xiao, X. et al. Freestanding mesoporous VN/CNT hybrid electrodes for flexible all-soli-state supercapacitors, *Adv. Mater.* **25**, 5091-5097 (2013).

6 Tinga, Y. J.; Lianb, K. & Kherani, N. Fabrication of titanium nitride and molybdenum nitride for supercapacitor electrode application, *ECS Transactions*, **32**, 133-139 (2011).

7 Su, F. et al. Preparation and characterization of highly ordered graphitic mesoporous carbon as a Pt catalyst support for direct methanol fuel cells. *Chem. Mater.* **17**, 3960-3967 (2005).

8 Cao, X. Y. et al. Quantitative investigation on the effect of hydrogenation on the performance of MnO_2/H-TiO_2 composite electrodes for supercapacitors. *J. Mater. Chem. A*, **3**, 3785-3793 (2015).
